# Intraocular pressure-lowering effects of Ripasudil: a potential outcome marker for Trabeculotomy

**DOI:** 10.1186/s12886-019-1253-4

**Published:** 2019-12-02

**Authors:** Erina Goda, Kazuyuki Hirooka, Kazuhiko Mori, Yoshiaki Kiuchi

**Affiliations:** 1 Department of Ophthalmology and Visual Science, Graduate School of Biomedical Sciences, Hiroshima University, 1-2-3 Kasumi, Minami-Ku, Hiroshima, 734-8551 Japan; 2 Department of Ophthalmology, Kagawa University Faculty of Medicine, Kagawa, Japan; 3 Department of Ophthalmology, Kyoto Prefectural University of Medicine, Kyoto, Japan

**Keywords:** Ripasudil, Trabeculotomy, Glaucoma

## Abstract

**Background:**

To examine the use of ripasudil as a trabeculotomy outcome marker in patients with primary open-angle glaucoma (POAG).

**Methods:**

Between May 2015 and December 2018, 35 eyes underwent trabeculotomy and were postoperatively followed for over 3 months. Ripasudil was defined as effective if drug administration resulted in a greater than 10% reduction in intraocular pressure (IOP). Patients were divided into effective (effective group) or non-effective (non-effective group) ripasudil administration groups. The need for additional glaucoma surgery or an IOP ≥ 21 mmHg indicated surgical failure. In both groups, a Kaplan-Meier survival-analysis was used to evaluate success probabilities related to postoperative IOP levels.

**Results:**

Effective IOP reduction occurred in 14 of 35 eyes after ripasudil administration, which was shown by a decrease of more than 10%. Postoperatively, both groups exhibited significant reductions of IOP and antiglaucoma medication use for up to 24 months. At 12 and 24 months after trabeculotomy, probabilities of success in the effective vs. non-effective group were 100% vs. 94.7 and 100% vs. 75.4%, respectively (*P* = 0.14).

**Conclusions:**

Trabeculotomy is effective for achieving an IOP < 21 mmHg in ripasudil effective POAG eyes. Examination of ripasudil’s IOP-lowering effects may be useful in predicting surgical outcomes after trabeculotomy.

## Background

Increases in outflow resistance can cause elevated intraocular pressure (IOP). There are two aqueous humor outflow pathways, with the aqueous primarily flowing through the trabecular meshwork and Schlemm’s canal to the episcleral vein. However, there is also an auxiliary uveoscleral pathway that passes through the iris root and ciliary muscle. Conventional pathways carry 80% of total aqueous humor out of the eye. Thus, in many types of glaucoma, an increased resistance to flow in these pathways is predominantly responsible for the elevated IOPs. Previous studies have examined Rho kinase inhibitors and reported finding an association with the alterations in the conventional outflow of aqueous humor [1, 2]. Inhibitor-caused increases in aqueous outflow have also been shown to lead to significant reductions in IOP [1, 2]. In 2014, the Rho kinase inhibitor ripasudil (K-115) was approved in Japan and has subsequently been shown to exhibit IOP-lowering effects in glaucoma patients [3–5]. The mechanism responsible for the IOP reductions by Rho kinase inhibitors involves the relaxation of the trabecular meshwork, with disruption of the action bundles leading to expansion of the intertrabecular space [1, 6].

Trabeculotomy performed in glaucoma patients reduces the IOP by decreasing the aqueous flow resistance by cleavage of the trabecular meshwork and inner walls of Schlemm’s canal [7]. Tanihara et al. examined eyes with primary open-angle glaucoma (POAG) and reported that success probability at 1 year after the surgery was 76.4% [7]. In other words, more than 20% of eyes exhibit a poor response to IOP reductions at 1 year after surgery. Therefore, after trabeculotomy, it is important to be able to determine clinical outcome markers of success for the procedure. Thus, the aim of the present study was to examine POAG patients and the potential use of ripasudil as an outcome marker of trabeculotomy.

## Methods

### Patient selection and surgical procedures

Between April 2015 and March 2018, this retrospective study examined eyes undergoing trabeculotomy treatments. All procedures and follow-ups took place at Kagawa University Hospital or Kyoto Prefectural University Hospital, Japan. If both eyes were subject to the treatments, only patient data from the first eye operated on was selected and used for the study. The Institutional Review Board of the Kagawa University Faculty of Medicine or Kyoto Prefectural University approved the study protocol. In accordance with the principles outlined in the Declaration of Helsinki, all subjects provided written informed consent in addition to the standard consent for surgery prior to their enrollment and participation in the research study.

Patients enrolled in the study had open-angle, glaucomatous visual field defects and a history of IOPs greater than 22 mmHg. To be defined as having a glaucomatous visual field, patients were required to have a glaucoma hemifield test outside of the normal limits. Inclusion criteria also required patients to be 20 years of age or older, and have a preoperative IOP of more than 22 mmHg despite the administration of the maximum tolerated medical therapy. Patients were excluded if they had any history of previous glaucoma surgery or significant ocular diseases, or if up to 3 months before the trabeculotomy they had undergone any previous intraocular surgery.

The trabeculotomy technique used in this study has previously been described [7]. Briefly, a one-half thickness scleral flap (approximately 4 × 4 mm) was created after the conjunctival incision at the corneal limbus. Inside the initial flap, a 4/5 thickness 3.5 × 3.5 mm parabolic flap was then dissected. After identification of Schlemm’s canal, a razor blade was used to cut the outer wall with the tissue excised using fine scissors. In both ends of the opened canal, U-shaped probes were inserted and rotated 90 degrees against the trabecular meshwork. By rotating the probes, this made it possible to create a 120° opening of the trabecular meshwork. After removed inner scleral flap, the outer scleral flap was sutured using 5 monofilament 10–0 nylon sutures. At the edge of the incision, the conjunctiva was closed using 8–0 silk sutures. At the end of the surgical procedure, we instilled corticosteroid/antibiotic ointment, followed by the application of a sterile eye patch and shield.

For the phacoemulsification procedure, the intraocular lens was placed into the capsular bag via the use of a 2.4 mm temporal clear cornea incision. Patients were treated with topical corticosteroid after the surgery (four times daily) in conjunction with the administration of an antibiotic for four weeks.

### Main outcome measure

With the exception of ripasudil, all patients enrolled in the study had been previously receiving the maximal tolerated medical therapy. Ripasudil was administered before surgery for at least two months [8]. After ripasudil was administered, we examined patients every month. We thus evaluated IOP at least twice after administration of ripasudil. We compared the average IOP before (twice) and after (twice) administration of ripasudil. Ripasudil was deemed as being effective if there was greater than a 10% reduction in the IOP after the ripasudil administration. Patients were subsequently divided into the effective (effective group) or non-effective (non-effective group) ripasudil administration groups. Patients who required additional glaucoma surgery or had an IOP ≥ 21 mmHg (criterion A) and ≥ 17 mmHg (criterion B) with or without ocular hypotensive medications at two consecutive visits were defined as surgical failures. A Goldmann applanation tonometer was used to examine the IOP. IOP was measured in the morning. Due to the occurrence of postoperative IOP fluctuations after trabeculotomy, we did not consider IOPs that met this criterion for up to 3 months after the surgery to be surgical failures [7].

### Statistical analysis

All statistical analyses were performed using SPSS for Windows (SPSS Inc., Chicago, IL). Comparisons between the effective and non-effective groups for the clinical characteristics were performed using a Student’s *t*-test for continuous variables and a chi-square test for categorical variables. The Kaplan-Meier survival curve and the log-rank test were used to compare the outcomes between the effective and non-effective groups. Cox proportional hazards regression model analysis was used to examine the predictive value of significant factors. The following factors were tested for associations with surgical failure: age, preoperative IOP, previous cataract surgery, combined cataract surgery and effectiveness of ripasudil. *P* < 0.05 was considered statistically significant. All statistical values are presented as the mean ± standard deviation (SD).

## Results

A total of 35 patients (35 eyes) met the inclusion criteria and were enrolled in the study. After administration of ripasudil, 14 of 35 eyes exhibited effective IOP reduction, which was defined as a decrease of more than 10% (effective group). Mean age was 59.4 ± 19.4 years and 62.6 ± 13.3 years in the effective and non-effective groups, respectively (*P* = 0.57). No significant differences were noted for gender (*P* = 1.00), preoperative IOP (*P* = 0.14), or the number of glaucoma medications (*P* = 0.53). Characteristics for both the effective and non-effective groups are listed in Table 1. The number of combined cataract surgeries in the effective and non-effective groups were 8 (57%) and 15 (71%), respectively (*P* = 0.66). The side effects noted included conjunctival hyperemia in 2 patients and blepharitis in 6 patients.

**Table 1:** Clinical characteristics

	Effective group (*n* = 14)	Non-effective group (*n* = 21)	*P* value
Age (years)	59.4 ± 19.4	61.2 ± 13.2	0.76
Gender (M/F)	6/8	9/12	1.00
IOP before administration of ripasudil (mmHg)	22.8 ± 3.7	21.4 ± 4.4	0.33
IOP after administration of ripasudil (mmHg)	19.0 ± 3.5	23.4 ± 8.0	0.10
Refractive error (D)	−4.6 ± 4.7	− 4.7 ± 4.9	0.92
Axial length (mm)	24.6 ± 1.7	25.5 ± 3.2	0.44
Central corneal thickness (m)	522.7 ± 31.9	530.1 ± 48.6	0.61
Preoperative IOP (mmHg)	27.4 ± 6.2	24.8 ± 3.9	0.14
Lens status			1.00
Phakia	12	18	
Pseudophakia	2	3	
HFA30–2 MD (dB)	−7.4 ± 4.1	−12.2 ± 6.4	0.03
Glaucoma medications	3.2 ± 2.0	3.4 ± 1.5	0.73
PGA + β blocker + CAI	3	6	
PGA + β blocker + CAI + brimonidine	2	7	
PGA + CAI + brimonidine	0	0	
PGA + CAI	0	1	
β blocker + CAI + brimonidine	0	0	
Others	9	7	

M; male, F; female, IOP; intraocular pressure, D; diopter, HFA; Humphrey field analyzer, MD; mean deviation

PGA; prostaglandin analogue, CAI; carbonic anhydrate inhibitor

**Fig. 1:**
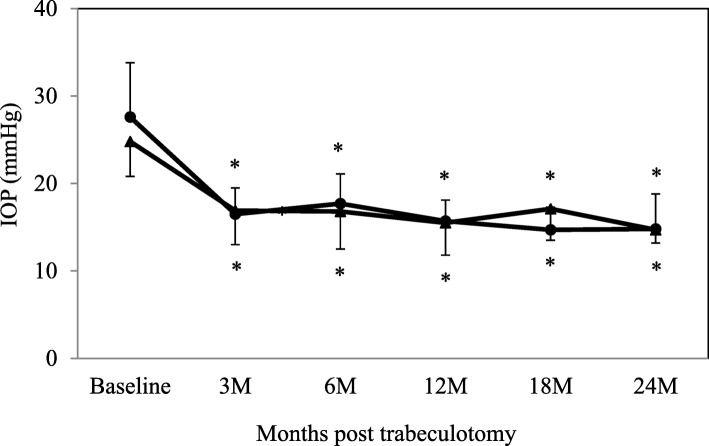
Mean intraocular pressure following trabeculotomy in the effective and non-effective groups. Intraocular pressure was significantly reduced in both groups compared with baseline. *: *P* < 0.05 compared with baseline. ●: effective group, ▲: non-effective group

**Fig. 2:**
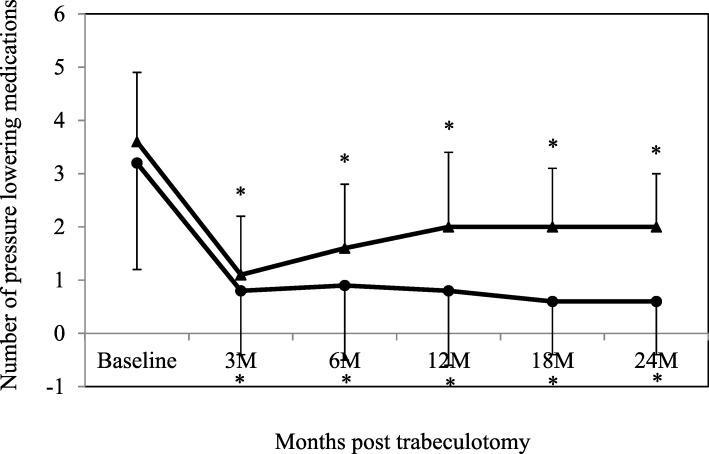
Mean antiglaucoma medications following trabeculotomy in the effective and non-effective groups. Antiglaucoma medications were significantly reduced in both groups compared with baseline. *: *P* < 0.05 compared with baseline. ●: effective group, ▲: non-effective group

**Fig. 3:**
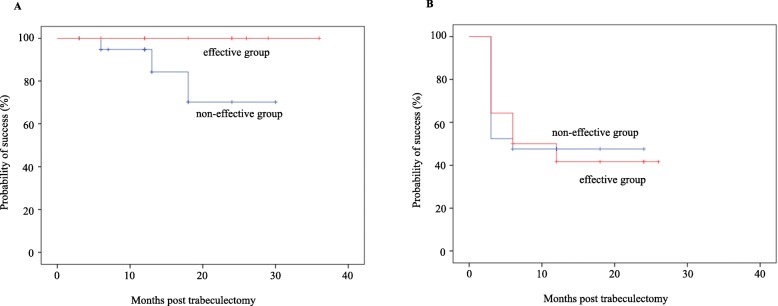
Kaplan-Meier survival curve of surgical outcomes in ripasudil effective vs. ripasudil non-effective eyes in criteria A (**a**) and B (**b**). No significant differences were noted for the cumulative probability of success between effective and non-effective groups (A: *P* = 0.14, B: *P* = 0.91)

The mean follow-up period was 19 months (range: 3–36 months) in the effective group, while it was 14 months (range: 3–30 months) in the non-effective group. After the surgery, both groups exhibited significant decreases in the IOP and in the number of antiglaucoma medications (Figs. 1 and 2). The mean IOP in the effective group was 27.4 ± 6.2 mmHg (*n* = 14) at baseline, while it was 16.5 ± 3.0 (n = 14), 17.7 ± 3.4 (*n* = 12), 15.7 ± 2.4 (n = 12), 14.7 ± 2.6 (*n* = 7), and 14.8 ± 4.0 mmHg (*n* = 6) at 3, 6, 12, 18, and 24 months, respectively. In the non-effective group the IOP was 24.8 ± 4.0 mmHg (*n* = 21) at baseline, while it was 16.9 ± 3.9 (n = 21), 16.8 ± 4.3 (*n* = 20), 15.5 ± 3.7 (*n* = 15), 17.1 ± 3.6 (*n* = 8), and 14.7 ± 1.5 mmHg (*n* = 3) at 3, 6, 12, 18, and 24 months, respectively. Figure 3 shows the Kaplan-Meier survival-curve analysis of the effective and non-effective groups. At 12 and 24 months after trabeculotomy, the probability of success in the effective and non-effective groups was as follows: 100% vs. 94.7 and 100% vs. 75.4%, respectively (*P* = 0.14) using criterion A and 41.7% vs. 47.6 and 41.7% vs.47/6%, respectively (*P* = 0.91), using criterion B. Three patients showed failure in criterion A. All had IOP ≥ 21 mmHg and underwent additional glaucoma surgery.

The mean number of glaucoma medications in the effective group was significantly decreased compared with the non-effective group at the final visit (Table 2). Postoperative complications were similar between groups (Table 3). Risk factors for failure were identified using cox regression analysis. However, we could not find any factors affecting surgical outcome in the current study (Table 4).

**Table 2:** Postoperative clinical data

	Effective group (*n* = 14)	Non-effective group (*n* = 21)	*P* value
Final MD of HFA30–2 (dB)	−8.1 ± 4.8	−11.8 ± 5.4	0.09
Glaucoma medications	1.0 ± 1.3	2.0 ± 1.3	0.04
PGA + β blocker + CAI	1	6	
PGA + β blocker + CAI + brimonidine	1	1	
PGA + β blocker	2	0	
PGA + CAI	1	0	
PGA	1	2	
β blocker + CAI	0	5	
CAI	0	1	

HFA; Humphrey field analyzer, MD; mean deviation, PGA; prostaglandin analogue,

CAI; carbonic anhydrate inhibitor

**Table 3:** Postoperative complications

	Effective group (n = 14)	Non-effective group (n = 21)	*P* value
Hyphema with niveau formation	2 (14.3%)	7 (33.3%)	0.21
Transient IOP elevation > 30 mmHg	4 (28.6%)	6 (28.6%)	0.48

IOP; intraocular pressure

**Table 4:** Cox regression analysis of various factors as a risk for surgical failure

Factors	Risk Ratio	Hazard Ratio (95% CI)	*P* value
Age (per year)	1.01	0.972–1.042	0.73
Preoperative IOP (per mmHg)	1.03	0.951–1.109	0.50
Previous cataract surgery	2.90	0.587–14.298	0.19
Combined cataract surgery	2.09	0.648–6.763	0.22
Effectiveness of ripasudil	0.72	0.332–1.578	0.42

CI; confidence interval, IOP; intraocular pressure

## Discussion

This study examined the trabeculotomy success rates for ripasudil effective and non-effective eyes. Although there was not a significantly higher cumulative probability of success after the trabeculotomy for the ripasudil effective eyes compared to the non-effective eyes, at 24 months after surgery the success rate was 100% for the effective group using criteria A.

Dannheim reported that IOP levels in 60% of 100 eyes with POAG were controlled below 24 mmHg without any administration of medication [9]. Tanihara et al. examined eyes with POAG and found that the probability of success (less than 20 mmHg) was 76.4% after 1 year [7]. Iwao et al. also examined POAG patients and found that at 1 year after trabeculotomy, the probability of success (less than 21 mmHg) was 73.2% [10]. Even in the non-effective group, the success rate at 12 months (94.7%) after trabeculotomy seemed to be better in the current study than in the previous study [7, 9, 10]. One possible explanation of the better surgical outcome in the current study was that we removed the inner scleral flap in the surgical technique. However, when discussing trabeculotomies, one of the most important points involves the indications. Thus, the question that needs to be answered is what can be used to identify cases for which trabeculotomy should be the preferred procedure?

Tanihara et al. previously reported finding a poor prognosis in eyes with POAG or exfoliation glaucoma when patients had higher preoperative IOPs [7]. In contrast, Iwao et al. examined steroid-induced glaucoma patients and reported that higher preoperative IOPs were not a prognostic factor for trabeculotomy surgical failures [10]. In fact, prognostic factors for trabeculotomy surgical failures have yet to be definitively identified even when other types of glaucoma are included [10]. Furthermore, other studies have reported that induced changes of the trabecular meshwork cellular activities are associated with the IOP-lowering effect of the Rho kinase inhibitor in animals and perfusion organ culture studies [1, 6]. As relief of outflow resistance in the trabecular meshwork is the primary target of trabeculotomies attempting to reduce the IOP, the effectiveness of the surgery in the ripasudil effective eyes could be due to the consistency between the surgical target and the modulating lesion.

According to previous Japanese patients who were already on maximum medical therapy, IOP decreased from 2.6 to 3.1 mmHg or approximately 15–16% from baseline after administration of ripasudil [5, 11–13]. We therefore defined a greater than 10% reduction in IOP after ripasudil administration as indicating effectiveness.

Phacotrabeculotomy is more effective than trabeculotomy alone in lowering IOP in POAG. The 3-year success probability of phacotrabeculotomy was 90.8%, while the probability for trabeculotomy alone was 62.7% [14]. The number of combined cataract surgeries in the effective (57%) and non-effective (71%) groups were similar in the current study.

In order to safely achieve IOP reduction without having to use the more risky bleb-based surgical procedures, studies have focused on developing a minimally invasive glaucoma surgery (MIGS) technique. In some of these approaches, it proved possible with little or no actual tissue removal to achieve trabecular bypass and increase the trabecular outflow, while other approaches utilized small-diameter shunts in order to facilitate aqueous humor flow across the trabecular meshwork [15]. The IOP was also lowered when using canal-based MIGS, which was able to improve the aqueous flow through a diseased trabecular outflow pathway that subsequently emptied into episcleral and conjunctival veins. Therefore, the ripasudil effective eye should also be viewed as a MIGS outcome marker.

Fellman et al. examined characteristics of an episcleral venous fluid wave (EVFW) and suggested that these could be used to predict trabeculotomy surgical outcomes [16]. However, EVFW can only be evaluated during the actual surgery. In contrast, it may be possible to preoperatively determine what the effective IOP reduction will be after ripasudil administration. Furthermore, if surgical outcomes can be predicted before surgery, this makes it possible to create a much more precise informed consent for patients prior to the surgery.

Limitations of the present study include first, there was only a small number of subjects examined in the study. Thus, a further study with a larger number of subjects will need to be undertaken to address this issue. Second, the present conclusions were not based on a long follow-up period. Therefore, studies with longer follow-ups will need to be performed in order to confirm the potential outcome marker of trabeculotomy.

## Conclusions

In conclusion, this study demonstrated that trabeculotomy success probabilities with IOP levels below 21 mmHg were 100% in ripasudil effective eyes. Thus, ripasudil could potentially be used as a trabeculotomy outcome marker in POAG patients. Furthermore, there is a lower likelihood that ripasudil effective eyes will need to undergo additional glaucoma surgery in the future. However, non-responsiveness to ripasudil does not indicate poor future response to trabeculotomy.

## Data Availability

The datasets used and analyzed during the current study are available from the corresponding author on reasonable request.

## Abbreviations

IOP: Intraocular pressure
MIGS: Minimally invasive glaucoma surgery
POAG: Primary open-angle glaucoma,
